# Endoscopic diagnosis and screening of Barrett's esophagus: Inconsistency of diagnostic criteria between Japan and Western countries

**DOI:** 10.1002/deo2.73

**Published:** 2021-11-15

**Authors:** Norihisa Ishimura, Eiko Okimoto, Kotaro Shibagaki, Shunji Ishihara

**Affiliations:** ^1^ Second Department of Internal Medicine Shimane University Faculty of Medicine Shimane Japan; ^2^ Division of Gastrointestinal Endoscopy Shimane University Hospital Shimane Japan

**Keywords:** Barrett's esophagus, esophageal adenocarcinoma, endoscopic diagnosis, guidelines, intestinal metaplasia

## Abstract

Barrett's esophagus (BE) is an endoscopically identifiable premalignant condition for esophageal adenocarcinoma (EAC). To diagnose BE precisely, careful inspection of the anatomic landmarks, including the esophagogastric junction and the squamocolumnar junction is important. The distal end of the palisade vessels and the proximal end of the gastric folds are used as the landmark of the esophagogastric junction in endoscopic diagnosis, with the latter solely used internationally, except in some Asian countries, including Japan. In addition, the diagnostic criteria adopted internationally for BE are inconsistent, particularly between Japan and Western countries. Recently updated guidelines in Western countries have included length criteria, with a 1‐cm threshold of columnar epithelium by endoscopic observation and/or histologic confirmation of the presence of specialized intestinal metaplasia. Since BE is endoscopically diagnosed at any length without histologic assessment in Japan, the reported prevalence of short‐segment BE is very high in Japan compared with that in Western countries. Although guidelines on screening exist for BE, the current strategies based on the presence of chronic gastroesophageal reflux disease with multiple risk factors may miss the opportunity for early detection of EAC. Indeed, up to 40% of patients with EAC have no history of chronic gastroesophageal reflux disease. To discuss BE on the same footing worldwide, standardization of diagnostic criteria, screening indication, and establishment of effective techniques for detecting dysplastic lesions are eagerly awaited. Japanese guidelines for BE should be revised regarding the length criteria, including the minimum length and long‐segment BE, in line with the recently updated Western guidelines.

## INTRODUCTION

The geographic variation in esophageal cancer incidence differs substantially between the 2 main histologic subtypes, esophageal squamous cell carcinoma (ESCC) and esophageal adenocarcinoma (EAC).^1^, ^2^, ^3^ ESCC is the predominant subtype in Asia, including Japan, while EAC is the predominant subtype in many Western regions, including Europe, North America, and Australia, with a rapidly increasing incidence noted in the last four decades.^2^, ^4^ Although the incidence of ESCC remains high, accounting for approximately 90% of all esophageal cancer cases in Japan, the incidence of EAC was reported to have increased steadily, more than threefold, for the past two decades according to the comprehensive registry of esophageal cancer in Japan.^5^, ^6^, ^7^ Barrett's esophagus (BE) is the only known premalignant condition for EAC, characterized by the replacement of normal stratified squamous epithelium with columnar metaplasia in response to chronic gastroesophageal reflux.^8^, ^9^, ^10^

Early detection of precancerous or neoplastic changes in Barrett's mucosa is of utmost importance because of the high mortality rate of EAC, which has a 5‐year overall survival rate of less than 25%.^11^, ^12^, ^13^ Therefore, sensitive and accurate diagnosis of BE is an important issue in clinical practice. For detection of BE, endoscopy is the best diagnostic tool, with histopathological confirmation used in an adjunctive manner. To standardize and improve clinical practice, multiple guidelines have been published, though the definition of BE and the strategy of BE management have yet to be standardized universally and internationally.^14^ Here, we present a review of the endoscopic diagnosis and screening of BE, focusing on the inconsistency of diagnostic criteria, especially between Japan and Western countries.

## MULTIPLE GUIDELINES ON BE

In Japan, the Japanese Esophageal Society (JES) definition of BE is well accepted and broadly used in the clinical setting.^15^ The Japanese Society of Gastroenterology briefly addressed BE in their recommendations for gastroesophageal reflux disease (GERD) published in 2015.^16^ The Asian Pacific Association of Gastroenterology updated its consensus statement on proton pump inhibitor‐refractory GERD and BE based on Asia‐Pacific data.^17^ In Europe, the European Society of Gastrointestinal Endoscopy (ESGE) published a position statement in 2017 that resulted from the consensus of four recent national guidelines.^18^ However, the British Society of Gastroenterology guidelines still differ from the ESGE guidelines on several domains of BE definition and management.^19^, ^20^ In the USA, three gastroenterological societies have contributed recommendations on the diagnosis and management of BE. The American Society of Gastrointestinal Endoscopy (ASGE) published recommendations in 2012 and recently updated those on BE screening, surveillance, and treatment (2017, 2018, and 2019).^21^, ^22^, ^23^ The American Gastroenterological Association has recently (2020) revised its previously published guidelines (2011 and 2016).^24^, ^25^, ^26^ In addition, the American College of Gastroenterology updated its guidelines in 2015.^27^ All of the current guidelines agree that BE should be diagnosed upon extension of the columnar epithelium into the distal esophagus. However, some controversy remains regarding the length and histologic criteria for BE diagnosis.^14^, ^28^

## DIAGNOSIS OF BE

**FIGURE 1:**
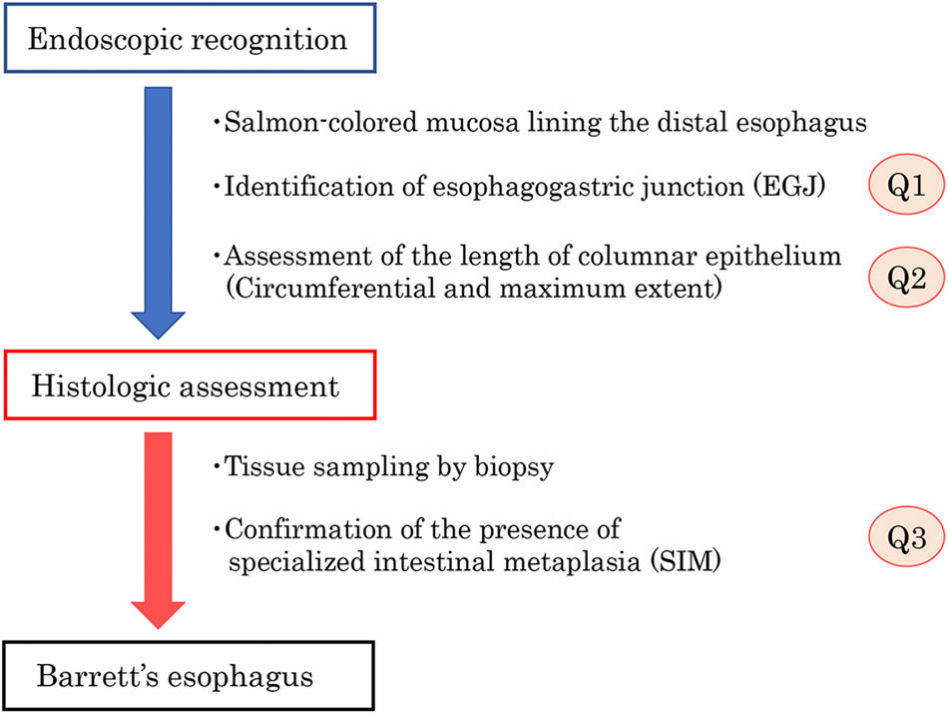
Diagnostic algorithm for Barrett's esophagus. When salmon‐colored mucosa lining the distal esophagus is observed on endoscopy, identification of the esophagogastric junction (EGJ) is the first step in the diagnosis of Barrett's esophagus (BE). Next is an assessment of the length of the columnar‐lined esophagus according to the Prague C&M criteria. Then, tissue sampling by biopsy and histologic assessment is conducted. Although the diagnosis of BE seems straightforward, there continues to be a debate in terms of endoscopic and histologic criteria, raising three major questions (see text)

A diagnostic algorithm for BE is shown in Figure 1. Three major issues remain to be resolved in terms of the endoscopic and histologic diagnosis of BE as follows: Q1) What is the optimal landmark for the esophagogastric junction (EGJ)? Q2) What are the optimal length criteria for BE? Q3) What is the optimal histologic diagnosis of BE? Each of these issues is discussed in greater detail below.

### What is the optimal landmark for the EGJ?

BE is usually diagnosed by endoscopy to detect salmon‐colored columnar mucosa in the distal esophagus and confirmed by pathology. Therefore, the endoscopic recognition of the key anatomic landmarks of the EGJ is the first step in the diagnosis of BE. For identification of the EGJ, the distal end of the esophageal palisade vessels and the proximal end of the gastric mucosal folds are used as a landmark, with the latter widely adopted in most countries. In contrast, the distal end of the palisade vessels is mainly used as a landmark of the EGJ in some Asian countries, including Japan, and the proximal end of the gastric folds is used as the EGJ if the palisade vessels cannot be clearly identified.^15^, ^29^, ^30^

**FIGURE 2:**
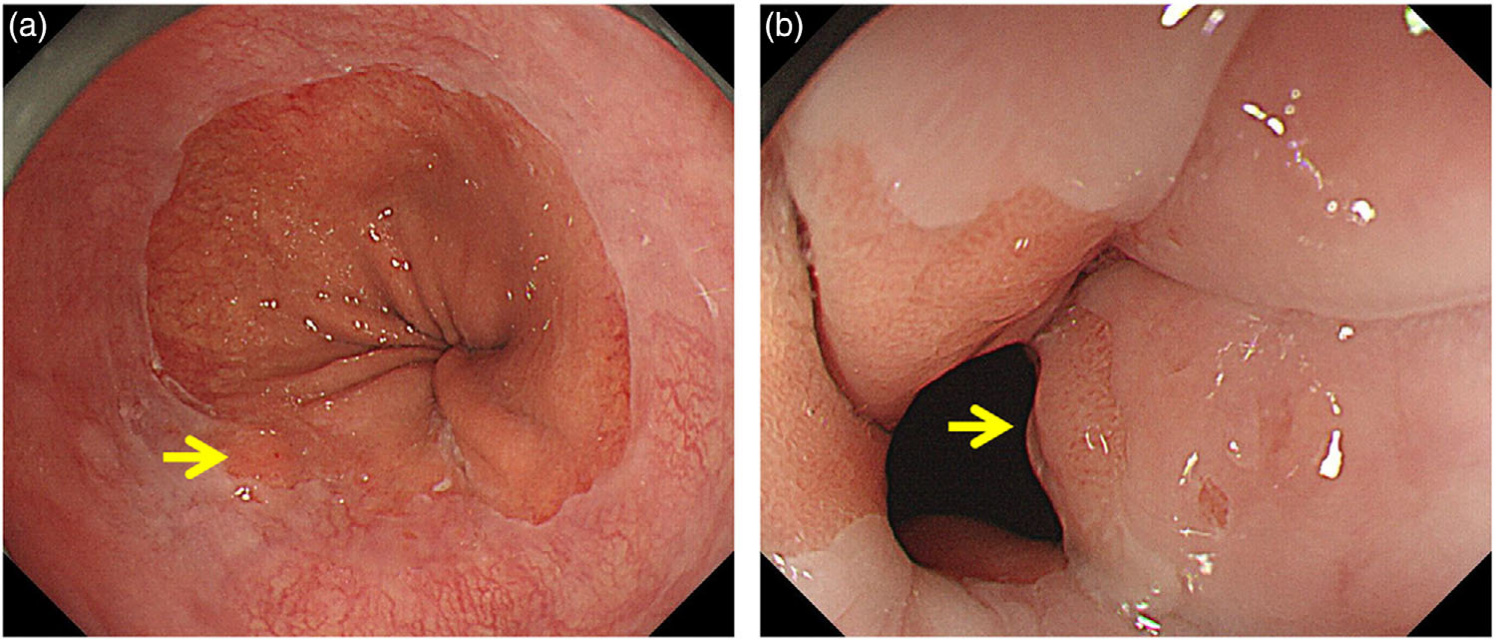
Endoscopic observation of short‐segment Barrett's esophagus. (a) Unsedated condition with air inflation via endoscopy. Palisade vessels and a dysplastic lesion (arrow) can be clearly detected by air inflation with cooperated deep breathing in the unsedated condition. (b) Under conscious sedation: those findings cannot be clearly detected in this condition

Palisade vessels, defined histologically as veins greater than 100 μm in size, run longitudinally in the mucosal layer within the lower esophageal sphincter, descending the submucosa once entering the cardia.^31^, ^32^ Therefore, the distal end of the palisade vessels coincides anatomically with the boundary between the esophagus and the stomach. Palisade vessels can be found easily on endoscopy when the lower esophagus is adequately distended by air through endoscopy with cooperated deep breathing (Figure 2a). However, they are sometimes difficult to identify owing to several factors, including insufficient extension under conscious sedation, mucosal inflammation, dysplastic changes, and the presence of a thick double muscularis mucosa (Figure 2b). Although some studies showed that the palisade vessels were visible to Western endoscopists in Western patients with BE,^33^, ^34^ current Western guidelines do not include the palisade vessels as a landmark of the EGJ.

**FIGURE 3:**
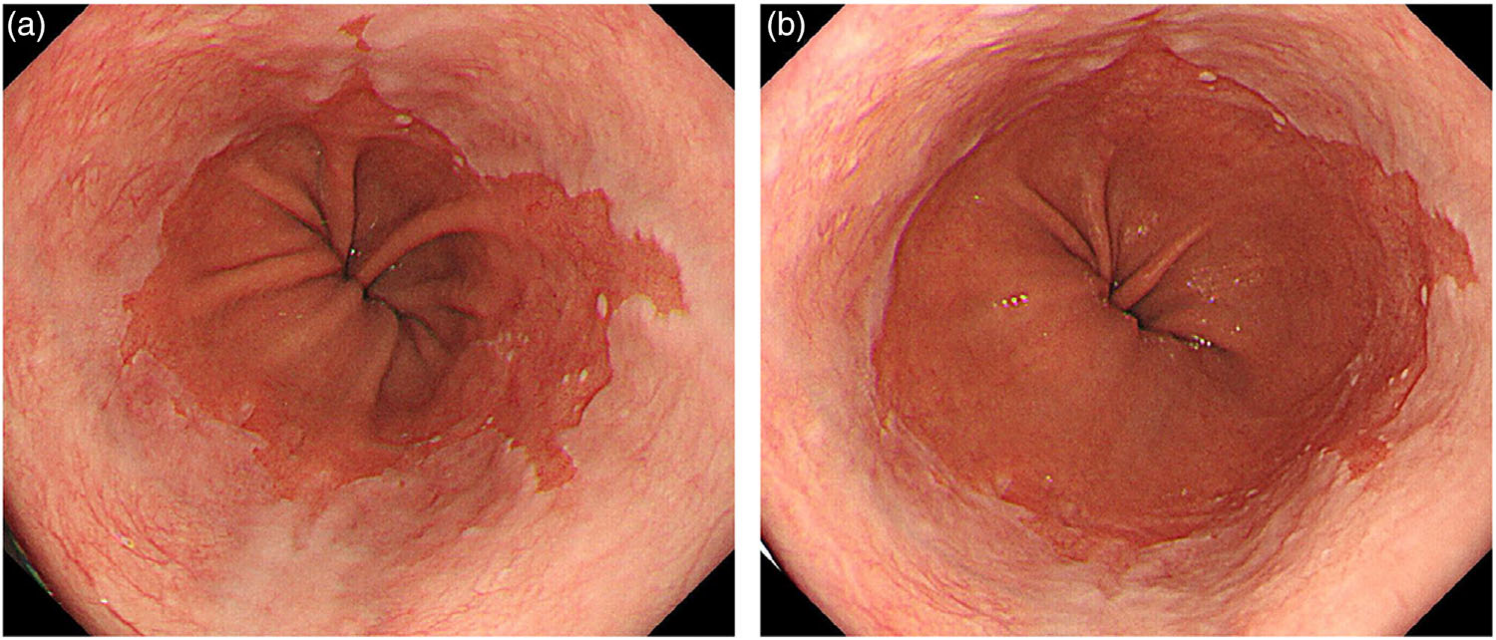
Endoscopic view of the esophagogastric junction. The position of the proximal extent of the gastric fold can easily change with the degree of air inflation. (a) Adequate condition, (b) excessive air inflation

The definition of the EGJ adopted in Western countries is the most proximal border of the gastric longitudinal fold.^14^ This definition also has potential drawbacks, as the position of the proximal end of the gastric fold can easily change with gut motility, respiration, and the degree of air insufflation with endoscopy (Figure 3).^35^ In addition, the proximal end of the gastric fold is difficult to identify in the presence of atrophic gastritis with *Helicobacter pylori* infection, which is more commonly observed in Japan. It is because of the difficulty of accurate identification of the EGJ even with the use of these landmarks, that the inter‐observer variability in the diagnosis of BE has been reported to be unacceptably high, especially in cases with BE length less than 1 cm.^35^, ^36^, ^37^ In a comparative study of the two landmarks, the proximal extent of the gastric folds was more accurate compared with the palisade vessels after a complete presentation of C&M criteria (C=circumferential length, M=maximal length) to endoscopists.^37^ Accordingly, the proximal extent of the gastric folds is still used as the EGJ in the most recent Western guidelines, despite poor concordance.^27^

Virtual chromoendoscopy (VC), including narrow‐band imaging (NBI), linked color imaging (LCI), and blue laser imaging, can accentuate surface mucosal patterns and vascular features without the use of stains or dyes, which is now available on many endoscopes and is useful for detecting gastrointestinal neoplastic lesions.^38^, ^39^, ^40^ In 2004, Hamamoto et al. reported the usefulness of NBI for visualization of the EGJ.^41^ More recently, endoscopic observation with LCI has been reported to improve the visibility of the palisade vessels as well as the area of Barrett's segment compared with that using white light imaging,^42^, ^43^ suggesting that VC may improve the diagnostic consistency to detect palisade vessels, especially for short‐segment BE (SSBE). Consequently, the distal end of the palisade vessels is thought to be more suitable to the anatomic criteria of the EGJ than the proximal end of the gastric folds.

### What are the optimal length criteria for BE?

The squamocolumnar junction or Z‐line is an endoscopically visible demarcation separating the esophageal squamous epithelium from the red‐colored columnar gastric epithelium. In BE, the salmon‐colored columnar epithelium extends proximally from the EGJ to the esophagus in a continuous manner. In Japan, BE is defined as any length of columnar mucosa in the distal esophagus (Table 1). However, recently updated guidelines in Western and Asia‐Pacific regions have included length criteria with a 1‐cm threshold of the columnar‐lined esophagus (CLE), because a CLE of less than 1 cm has high interobserver variability as well as a low risk of EAC.^17^, ^18^, ^20^, ^27^ If the length of the CLE is less than 1 cm, it is called an irregular Z‐line or specialized intestinal metaplasia (IM) of the EGJ in those guidelines, although the older American Gastroenterological Association and ASGE guidelines do not provide a length threshold.^23^, ^26^

**TABLE 1:** Diagnostic criteria for Barrett's esophagus

**Guideline (Area, published year)**	**EGJ landmark**	**Length**	**Histology**
JES (Japan, 2015)	1) Lower end of palisade vessels 2) Proximal end of gastric folds (when palisade vessels are not clear)	Any length	Columnar epithelium
APAGE (Asia‐Pacific, 2016)	Proximal end of gastric folds	≥1 cm^‡^	Columnar epithelium
BSG (UK, 2014)	Proximal end of gastric folds	≥1 cm^‡^	Columnar epithelium
ESGE (Europe, 2017)	Proximal end of gastric folds	≥1 cm^‡^	Intestinal metaplasia
ACG (USA, 2016)	Proximal end of gastric folds	≥1 cm^‡^	Intestinal metaplasia
AGA (USA, 2011)	Proximal end of gastric folds	any length	Intestinal metaplasia
ASGE (USA, 2012)	Proximal end of gastric folds	none	Intestinal metaplasia

^‡^ Endoscopists should utilize the Prague classification to describe what is seen in Barrett's segment.

Abbreviations: ACG, American College of Gastroenterology; AGA, American Gastroenterological Association; APAGE, Asian Pacific Association of Gastroenterology; ASGE, American Society of Gastrointestinal Endoscopy; BSG, British Society of Gastroenterology; EGJ, esophagogastric junction; ESGE, European Society of Gastrointestinal Endoscopy; JES, Japanese Esophageal Society.

Accordingly, the reported prevalence of BE based on endoscopy findings in Japan varies widely, with results ranging from 15% to 85.9%, in contrast to the range in Western countries of 5% to 20%.^44^, ^45^, ^46^, ^47^ Adachi et al. reported that the prevalence of BE with a length of less than 1 cm was 56.2%, while that with a length of ≥ 1 cm was 26.2% when LCI was used to determine the area of BE, as the distal end of the palisade vessels was easily visualized.^42^ We investigated the inter‐institutional variability in the diagnosis of BE at four different hospitals. Because of cases of over‐ and under‐diagnosis, we demonstrated that the variance was unacceptably large (17.2%–96.8%), and the diagnostic accuracy was inadequate, especially in cases with a BE length of less than 1 cm, suggesting that the minimum length of CLE should be defined in future criteria in Japan.^48^ Although most studies show that the incidence of EAC in BE of less than 1 cm in length is very low,^49^ Barrie et al. recently reported that almost 20% of all dysplasia in BE and EAC occurs within a centimeter of the EGJ, suggesting that all lengths of CLE above the EGJ should be recognized as BE and subjected to a thorough biopsy protocol.^50^

**FIGURE 4:**
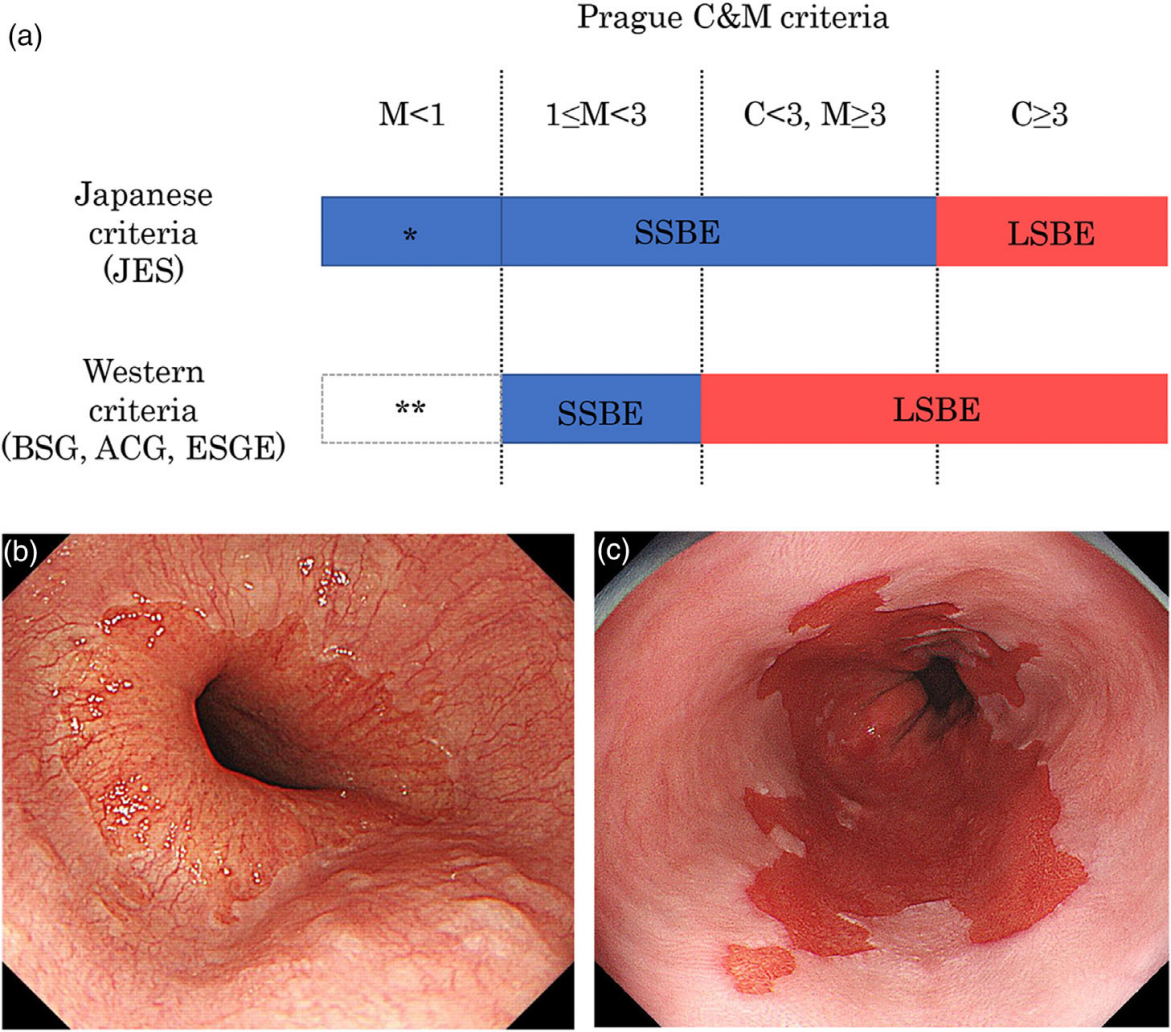
Diagnosis of Barrett's esophagus based on length. (a) In Japan, Barrett's esophagus (BE) is defined as any length of the columnar‐lined esophagus (CLE), while in recently updated Western guidelines, BE is defined as CLE with length ≥ 1 cm. In Japan, long‐segment BE (LSBE) is defined as the presence of circular CLE extending 3 cm or more, while in Western countries, LSBE is generally defined as CLE with maximal length greater than 3 cm. *CLE with length <1 cm is also termed as ultrashort‐segment BE (USSBE). **An irregular Z‐line or specialized intestinal metaplasia of the esophagogastric junction. (b) Endoscopic view of CLE with length <1 cm (M < 1). (c) Endoscopic view of CLE with maximal length greater than 3 cm (C <3, M ≥3).

Traditionally, BE is divided into long‐segment BE (LSBE, ≥3 cm) and SSBE (<3 cm) based on the length of columnar mucosa in the distal esophagus as assessed by endoscopy. Although not clearly defined in all guidelines, BE less than 1 cm in length is termed ultrashort‐segment BE (USSBE).^17^, ^51^ Since it is well understood that the risk of EAC increases with the length of BE,^52^, ^53^, ^54^ endoscopic inspections should receive more attention in patients with LSBE than in those with SSBE and USSBE. In Japan, LSBE is defined as the presence of circular Barrett's mucosa extending longitudinally for 3 cm or more, while SSBE is defined as the presence of circular Barrett's mucosa less than 3 cm in length or the presence of non‐circular Barrett's mucosa.^15^ In contrast, in other countries, LSBE is generally defined as Barrett's mucosa with a maximal length greater than 3 cm (Figure 4).^30^ To date, the risk of EAC arising from LSBE with and without circular Barrett's mucosa extending over 3 cm remains to be elucidated. Recently, a Japanese multicenter prospective cohort study showed that the incidence of EAC in patients with LSBE defined as Barrett's mucosa with maximal length greater than 3 cm was 1.2%/year, similar to the values in reports from Western countries.^55^ This suggests that the term LSBE should be used for Barrett's mucosa with maximal length greater than 3 cm for risk stratification based on length.

**FIGURE 5:**
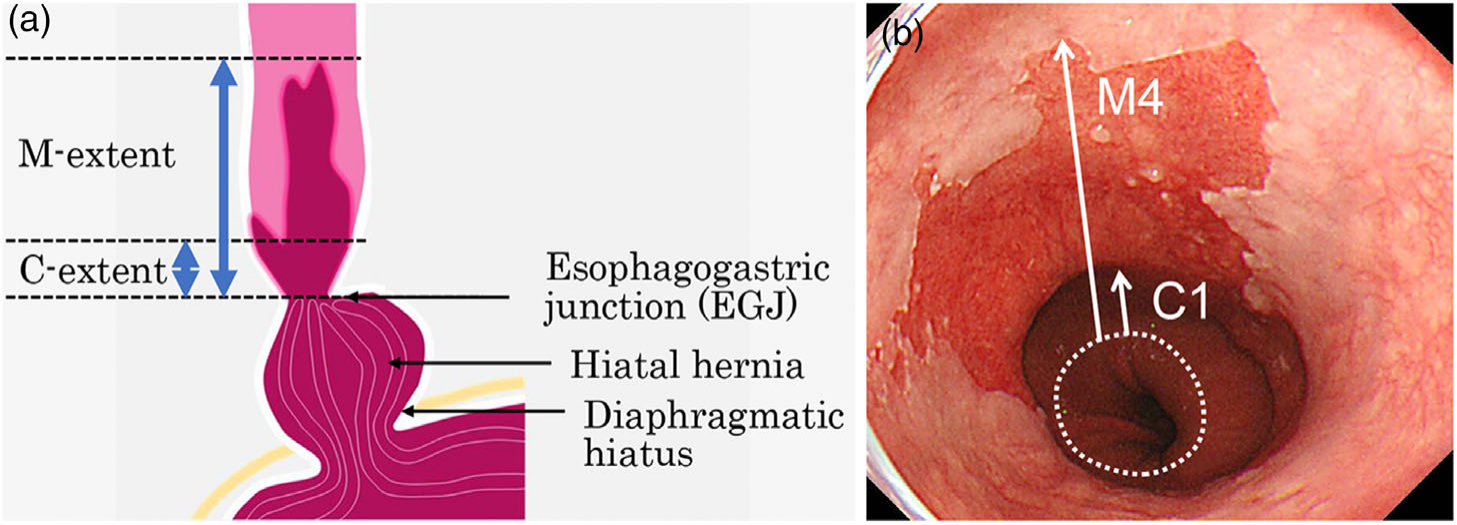
Endoscopic diagnosis based on Prague C&M criteria. (a) Endoscopic criteria for a diagnosis of Barrett's esophagus are described in the Prague classification. The distance from the esophagogastric junction (EGJ), defined as the proximal end of the gastric fold to the top of the circumferential columnar mucosa, is the C‐extent in centimeters, and the distance from the EGJ to the maximal extent of the columnar mucosa is the M‐extent in centimeters. (b) In this case, the Prague classification is C1M4.

Further assessment and documentation of the BE segment should include the Prague C&M criteria, describing the circumferential (C) and maximal (M) extent of BE (Figure 5).^36^, ^56^ This system classifies BE based on both the length of circumferential involvement and the maximal proximal extent of endoscopically visible columnar mucosa in the esophagus to simplify and standardize endoscopic characterization of the length and shape of BE. Because the increasing length of the BE segment is a known risk factor for the development of EAC, most recent guidelines recommend recording the length of BE in centimeters using these criteria.^10^, ^57^, ^58^, ^59^

### What is the optimal histologic diagnosis of BE?

The large number of studies demonstrating an oncogenic link between IM and EAC has led to the inclusion of the histologic confirmation of specialized IM, characterized by the presence of goblet cells for diagnosis of BE in most guidelines (Table 1).^26^, ^60^, ^61^ However, several studies showed that EAC may arise in the columnar mucosa without specialized IM.^62^, ^63^ In addition, specialized IM may be missed due to insufficient biopsy sampling, suggesting that specialized IM may not be essential for a BE diagnosis.

In contrast, the JES defines BE as any type of columnar epithelium continuous from the stomach, regardless of the presence of specialized IM.^15^ Histologically, at least one of the following findings must be observed: 1) proper esophageal gland ducts, 2) squamous island, and 3) double‐layer muscularis mucosa. Among these findings, squamous islands can be detected by endoscopy, and more effectively by NBI.^64^ Similar to the Japanese criteria, the Asian Pacific Association of Gastroenterology and British Society of Gastroenterology guidelines suggest that the presence of IM is not a prerequisite for the definition of BE,^20^ but should be taken into account when deciding the surveillance strategy.

At the initial diagnosis, the Seattle biopsy protocol, which entails four‐quadrant biopsies every 2 cm in addition to targeted biopsies of macroscopically visible lesions, is recommended if the columnar lining is greater than 1 cm above the EGJ in most Western guidelines.^49^, ^65^ However, the protocol is time‐consuming, practicing endoscopists have poor adherence to it, and there is an increased risk associated with the large number of biopsy samples required.^66^ Since the majority of BE is SSBE, targeted biopsies are the standard protocol for endoscopic observation in Japan.^30^

## SCREENING OF BE BY ENDOSCOPY

Most guidelines recommend against BE screening in the general population, but state that endoscopic screening can be considered in high‐risk individuals. The common risk factors include chronic GERD symptoms, age older than 50 years, white race, male sex, obesity, smoking history, and a first‐degree relative with BE or EAC.^67^ Consistently, Rubenstein et al. created a prediction tool incorporating GERD frequency, age, waist‐to‐hip ratio, and pack‐years of cigarette use, and the tool showed substantially improved prediction of the presence of BE compared with a model using GERD symptoms alone.^67^, ^68^ Although the threshold for the number of risk factors considered for screening varies among societies, all of those guidelines recommend endoscopy as the method for BE screening for high‐risk individuals (Table 2). In contrast, there is no recommendation for BE screening in Japanese guidelines, likely due to the low prevalence of EAC. However, due to the higher prevalence of gastric cancer, screening endoscopy has been widely executed with low cost for asymptomatic healthy subjects as a part of the comprehensive health check‐up to detect gastric cancer at the early stages. Therefore, most cases of BE are diagnosed incidentally on endoscopy findings obtained during a health check‐up, and most EACs are detected at an early stage.^5^

**TABLE 2:** Screening for Barrett's esophagus

	**JES**	**APAGE**	**BSG**	**ESGE**	**ACG**	**AGA**	**ASGE**
Recommendation	None	No value	Consider	Consider	Consider	Suggest	Consider
Threshold			≥3 risk factors	Long‐standing GERD + multiple risk factors	Males with chronic GERD + ≥2 risk factors	Multiple risk factors	Multiple risk factors
Risk factors			Age >50 years Male White race Chronic GERD Obesity Family history	factors Age >50 years Male White race Obesity Family history	Age >50 years White race Central obesity Smoking Family history	Age >50 years Male White race Chronic GERD Obesity Hiatal hernia	Age >50 years Male White race Chronic GERD Obesity Smoking Family history

Abbreviations: ACG, American College of Gastroenterology; AGA, American Gastroenterological Association; APAGE, Asian Pacific Association of Gastroenterology; ASGE, American Society of Gastrointestinal Endoscopy; BSG, British Society of Gastroenterology; EGJ, esophagogastric junction; ESGE, European Society of Gastrointestinal Endoscopy; GERD; gastroesophageal reflux disease; JES, Japanese Esophageal Society.

Screening of BE based on the presence of multiple risk factors, as described above, may miss the opportunity for early detection of EAC in a large number of asymptomatic patients. Although chronic GERD is thought to be the principal causal risk factor for EAC based on previous studies,^69^, ^70^ previous studies showed that up to 40% of patients with EAC had no history of chronic GERD.^71^, ^72^ Furthermore, sampling and diagnostic errors with inter‐variable pathological discrepancies result in reduced effectiveness of screening. According to the recent meta‐analysis by Tan et al., only 11.8% of patients with EAC had a prior BE diagnosis, though concurrent BE was found in up to 60% at the time of EAC diagnosis. In particular, up to 91% of all newly diagnosed patients with early‐stage EAC had BE on histopathology at the time of cancer diagnosis, and these findings raise the question of whether the population at risk of EAC is correctly identified and managed.^73^

## ENDOSCOPIC DETECTION OF DYSPLASTIC LESIONS

When BE is suspected on endoscopy, careful inspection should be conducted prior to obtaining biopsies to look for subtle visible abnormalities. Indeed, longer inspection time has been associated with a higher likelihood of diagnosing dysplastic lesions.^74^ Recently, the ESGE endorsed examination of the BE segment at a rate of 1 min per cm as a quality measure.^75^ Compared with standard‐definition white light, high‐definition white light improved dysplasia detection with an odds ratio of 3.27 (95% confidence interval, 1.27–8.40)^76^ and is recommended in current guidelines. To increase yield in the detection of dysplasia or early cancer, the ASGE recommends chromoendoscopy or VC in addition to white‐light endoscopy and biopsy specimens obtained using the Seattle protocol compared with white‐light endoscopy and biopsy specimens obtained using the Seattle protocol alone.^21^ Several advanced imaging modalities have been investigated to improve the detection and identification of early neoplastic lesions during surveillance endoscopy. Based on the ASGE meta‐analysis in 2016, only chromoendoscopy using acetic acid and VC using NBI met the ASGE Preservation and Incorporation of Valuable Endoscopic Innovations thresholds (sensitivity ≥90%, negative predictive value ≥98%, and specificity ≥80%).^77^ Indeed, NBI has been shown to increase the detection of dysplasia as well as to reduce the number of biopsies needed per patient.^78^, ^79^

During the endoscopic examination, particular attention should be paid to the right side of the esophagus extending from 12‐ to 6‐o'clock position, especially for SSBE, because EAC arising from SSBE is reported to be mainly located in the right side of the esophageal lumen likely due to potential inflammatory mechanisms for the circumferential predilection of EAC.^80^, ^81^, ^82^, ^83^ In contrast, the distribution of EAC arising from LSBE remains to be clearly elucidated.^84^ We previously showed that EAC at the EGJ in LSBE was frequently located on the right anterior wall, while EAC distant from the EGJ showed no characteristic circumferential distribution, suggesting that development of this type of lesion may be less affected by gastroesophageal reflux.^85^

To aid in the ability to identify dysplasia and cancer on NBI, the Barrett's International NBI Group has developed and validated an NBI classification system in patients with BE. The system, which includes the assessment under NBI with near‐focus imaging of the mucosal pattern and the vascular pattern as either regular or irregular, has greater than 85% accuracy and a high level of interobserver agreement.^86^ More recently, the JES classification of BE using magnification NBI was developed. This classification very simply categorizes most mucosal or vascular descriptions as regular for non‐dysplastic and irregular for dysplastic BE.^87^, ^88^

To reduce rates of underdiagnosed or undiagnosed neoplasia in the upper gastrointestinal tract, artificial intelligence (AI) has recently been introduced to assist endoscopists in the detection and diagnosis of upper gastrointestinal neoplasia, including esophageal cancer.^89^ To date, several reports have been published regarding the detection of neoplasia in BE using AI technology.^90^, ^91^, ^92^, ^93^, ^94^ A recent meta‐analysis by Arribas et al. showed that AI‐aided endoscopy can detect BE‐related neoplasia with high sensitivity (89%) and specificity (88%), indicating that the AI system is a promising tool to avoid missing neoplasia during endoscopy.^95^

## CONCLUSIONS

The incidence of EAC arising from BE has been increasing worldwide, and better strategies must be developed for the early detection and prevention of EAC. However, the definition of BE remains to be standardized universally and current strategies for BE screening are far from being satisfactory. Although endoscopy with biopsy continues to be the gold standard in a clinical setting, minimally invasive, non‐endoscopic technologies for BE have shown promise in recent clinical trials.^96^, ^97^ To discuss BE on the same footing, international standardization of diagnostic criteria, screening indications, and techniques, as well as a more personalized approach to surveillance, are eagerly awaited. Unfortunately, current Japanese guidelines for BE do not use the GRADE system and should be revised regarding the length criteria, in line with recently updated Western guidelines.

## CONFLICT OF INTEREST

Author Norihisa Ishimura is an associate editor of DEN Open. The rest of the authors do not have any conflict of interest.

## FUNDING INFORMATION

None
